# New insights into antigen specific immunotherapy for chronic myeloid leukemia

**DOI:** 10.1186/1475-2867-12-52

**Published:** 2012-12-15

**Authors:** Yangqiu Li, Chen Lin, Christian A Schmidt

**Affiliations:** 1 Institute of Hematology, Medical College, Jinan University, Guangzhou, 510632, China; 2 Key Laboratory for Regenerative Medicine of Ministry of Education, Jinan University, Guangzhou, 510632, China; 3 Department of Microbiology and Immunology, Medical College, Jinan university, Guangzhou, China; 4 Department of Hematology and Oncology, Ernst-Moritz-Arndt University Greifswald, Greifswald, Germany

**Keywords:** Chronic myeloid leukemia, Vaccine, BCR-ABL, Immunotherapy

## Abstract

Chronic myeloid leukemia (CML) is a stem cell disease in which BCR/ABL plays an important role as an oncoprotein and a molecular and immunogenic target. Despite the success of targeted therapy using tyrosine kinase inhibitors (TKIs), CML remains largely incurable, most likely due to the treatment resistance of leukemic stem cells. Several immunotherapies have been developed for CML in different stages and relapse after allogeneic stem cell transplantation. In the this review, several specific immunotherapeutic approaches for CML, including vaccination and adoptive cellular immunotherapy, are discussed along with results from clinical trials, and the value of such immunotherapies in the era of imatinib and leukemia-associated antigens (LAAs), which are capable of inducing specific T cell responses and are appropriate target structures for the immunological targeting of CML cells, are also summarized.

## Introduction

Chronic myelogenous leukemia (CML) is a clonal myeloproliferative hematopoietic stem cell disorder that is characterized by a t(9;22) translocation, which results in the expression of BCR-ABL fusion oncoproteins that are unique to the leukemic cells, necessary for oncogenesis, and potentially immunogenic [1].

The BCR-ABL tyrosine kinase inhibitor imatinib is highly effective for first-line CML treatment and is increasingly used in patients with residual disease or relapse after allogeneic hematopoietic stem cell transplantation (allo-HSCT). Despite the success of imatinib and other tyrosine kinase inhibitors (TKIs), CML remains largely incurable, and this is likely due to the treatment resistance of leukemic stem cells, which are responsible for rapid disease relapse after the discontinuation of therapy. How to treat patients with CML who are resistant to BCR-ABL tyrosine kinase inhibitors is an important and urgent issue for clinical hematology. Based on experimental research exploring the imatinib resistance mechanism in CML cells, second-generation TKIs were developed. Dasatinib and nilotinib, two newer drugs with higher potency than imatinib against BCR-ABL and activity against most imatinib-resistant BCR-ABL mutations, have demonstrated superior efficacy compared with imatinib for first-line chronic-phase CML treatment in randomized phase III trials [2,3]. However, because successful treatment of a portion of patients with CML using allo-HSCT suggests the importance of immune mechanisms in eliminating leukemic cells including leukemia stem cells, TKI administration or HSCT may be combined with vaccination to cure patients with CML [4].

The history of CML immunotherapetic strategies begins as early as 1975 when patients with CML received repeated intradermal BCG-cultured cell mixture injections or were vaccinated with BCG alone in a clinical immunotherapy trial, and data from cases in which intermittent busulfan therapy was used provided evidence suggesting that immunotherapy prolonged the unmaintained remission of one-third of patients [5]. Today, in the molecular biology and immunology era, increasing effective and specific immunotherapies involving vaccination or adoptive cellular immunotherapy are used.

## Immune status in CML

In patients with leukemia, T cell function becomes suppressed with disease progression. Such immune dysfunction, which has been demonstrated in many patients with leukemia, may be due to a disorder in the thymic output function, the abnormal expression of the T cell receptor (TCR) repertoire and, in part, abnormal TCR signal transduction, possibly through altered CD3 gene expression [6-12]. In de novo CML, decreased levels of recent thymic emigrants in CD4+ and CD8 + T cells may underlie the persistent immunodeficiency found in patients. Restricted TCR Vβ repertoire expression indicates T cell immunodeficiency in patients, although clonally expanded T cells suggest a specific immune response to leukemia-associated antigens [9,13]. A deficiency in the level of CD3 gene expression may be a characteristic of lower T cell activation [6-8]. The absence of the TCRζ chain not only influences the level of TCR expression on the cell membrane and the number of single positive (CD4+ or CD8+) circulating T cells, it also impairs the proliferative response and mature T cell activation level. T cells from patients with CML are functionally impaired, and this is indicated by decreased TCRζ chain expression [6,9,14].

Moreover, imatinib impairs CD8+ T cells specifically directed against leukemia-associated antigen function in vitro; therefore, clinical imatinib administration may result in reduction of the efficacy of the graft-versus-leukemia effect or other T-cell-based immunotherapies [15].

In contrast, it has been demonstrated that patients with CML possess T cells capable of recognizing autologous tumor cells, and clonally expanded T cells were identified in some TCR subfamilies in the peripheral blood of patients with CML, which display specific anti-leukemia cytotoxicity such as WT1 or BCR-ABL-specific cytotoxic T cells (CTLs), indicating that specific anti-leukemic T cells could be generated in vivo [13,16,17]. This finding suggested that the host could have a specific immune response to leukemia-associated antigens despite T cell immunodeficiency. Several clinical observations, which were supported by experimental data, indicate the presence of CML-specific T cells.

Leukemia-specific T cells are regularly detected in patients with CML and may be involved in the immunological control of the disease. However, recent findings demonstrated that leukemia-specific CTLs maintain only limited cytotoxic activity, do not produce interferon-γ or tumor necrosis factor-α, and do not expand after restimulation. Because CML-specific CTLs were characterized by the high expression of programmed death 1 (PD-1) and CML cells expressed PD-ligand 1 (PD-L1) [18], this phenomenon was found not only in a CML mouse model but in patients with CML as well [19]. PD-1 is another inhibitory T-cell receptor that is engaged by its two known ligands PD-L1 (also known as B7-H1 or CD274) and PD-L2 (also known as B7-DC or CD273) primarily within the tumor microenvironment, and increased PD-1 and PD-L1 expression appeared to increased immune suppressive signals, which was related to inhibition of the effector phase of T cell responses and reduced antitumor activity [20].

Increased understanding of the molecular biology and immunology of CML and highly specific immune responses will lead to novel and improved immunotherapeutic strategies for patients with CML.

## Vaccines

Despite the considerable successes that have been achieved for CML treatment, a cure for this disease can only be obtained via the current treatments in a minority of patients. During the past decade, considerable progress has been made in the understanding of the immunology of CML, which has raised hopes that this disease may be curable by supplementing the current targeted chemotherapy with immunotherapeutic approaches. More than ten small-scale clinical trials have been performed using experimental vaccines predominantly based on the p210 BCR-ABL fusion protein [21], and other attractive targets include the Wilms' tumor 1 (WT1) antigen and the PR1 epitope from proteinase 3, a granule protein overexpressed in CML. In clinical trials, peptide vaccination appears safe and undoubtedly results in clinical effects.

### BCR-ABL-derived peptide vaccines

Nearly all patients with CML express the BCR-ABL fusion product on their leukemia cells. The chimeric p210 BCR-ABL fusion protein, comprising products of either the b2a2 or b3a2 exon junction, represents a potentially immunogenic tumor-specific antigen. Despite the intracellular location of this oncogenic fusion protein, it has been shown that peptides derived from its junctional region can be recognized by human T cells obtained from patients with CML or normal donors and can elicit a BCR-ABL peptide-specific T cell immune response.

The first evidence of a cytolytic human immune response against CML BCR-ABL oncogene-derived peptides was described by Bocchia et al. who demonstrated that peptides derived from amino acid sequences crossing the b3a2 fusion breakpoint in p210 elicit class I restricted cytotoxic T cells and class II-mediated T cell proliferation, respectively, in vitro [22,23]. Thus, such sequences may comprise definitive tumor-specific antigens in a peptide-based vaccine and provide a rationale for developing peptide-based vaccines for CML.

The different BCR-ABL breakpoint peptide vaccines were evaluated in numerous clinic trials. Pinilla-Ibarz J et al. were the first to develop a BCR-ABL-derived peptide vaccination strategy [24]. The vaccine was well tolerated and elicited specific immune responses in patients with CML. In a phase II trial from the same group, 14 patients with chronic phase CML were vaccinated 5 times. Immunological and clinical effects were detected; however, CTLs were not identified [24,25]. Thus, in phase I and II trials, a tumor-specific BCR-ABL derived peptide vaccine could be safely administered to patients with chronic phase CML and elicit BCR-ABL peptide-specific CD4 immunity. However, CD8 responses were limited. Therefore, one strategy to circumvent this poor immunogenicity is to design synthetic immunogenic analog peptides that cross-react with native peptides (i.e., a heteroclitic response). A number of synthetic peptides derived from CML junctional sequences (i.e., p210/b3a2 or p210/b2a2) in which single and double amino acid substitutions were introduced at key HLA-A0201 binding positions were screened for eliciting HLA restricted, peptide-specific CTL responses using CD3+ T cells from several A0201 donors and patients with CML [26].

A significant BCR-ABL vaccination effect was described in a 63-year-old woman with CML relapse after interferon (IFN)-α treatment who achieved a complete cytogenetic response for 6 years. The patient was treated with a therapeutic vaccine comprising an immunogenic 25-mer b2a2 breakpoint-derived peptide (CMLb2a2-25) with binding properties for several HLA-DR molecules. After nine vaccine boosts, the patient developed an adequate b2a2-25 peptide-specific CD4+ T cell response, and the BCR-ABL1 transcript began declining in the peripheral blood. At the last evaluation i.e., 39 months since the vaccinations commenced, the patient is in complete molecular response with an undetectable level of BCR-ABL1 transcript in the peripheral blood and bone marrow. She continues to receive a vaccine boost every 3 months as her only treatment [27].

Recently, BCR-ABL peptides were used as combination immunotherapy in imatinib-treated patients with CML. BCR-ABL peptide vaccination may improve the control of CML, particularly in patients responding well to imatinib. A trial with 19 imatinib-treated patients with CML in the first chronic phase were vaccinated with BCR-ABL peptides spanning the e14a2 fusion junction, and 14 of the 19 patients developed T cell responses to BCR-ABL peptides. The development of an anti-BCR-ABL T cell response correlated with a subsequent decrease in BCR-ABL transcripts. Of the 14 patients in MCR at baseline, 13 developed at least a 1 log decrease in BCR-ABL transcripts [28]. Similar results were reported in a phase II trial in which 10 patients who had received imatinib for a median of 62 months were enrolled [29]. These data suggested that a vaccination-related transient disruption in immune tolerance may contribute to a reduction in BCR-ABL transcripts, and this BCR-ABL peptide vaccine may transiently improve the molecular response in a subset of patients with CML.

Although most patients with CML achieve clinically relevant hematologic and cytogenetic responses to imatinib, CML cells with a BCR-ABL mutation (T315I) confer drug resistance to imatinib, dasatinib and nilotinib treatment; therefore, the development of a vaccine expressing the T315I-mutated BCR-ABL antigen to stimulate an anti-BCR-ABL (T315I) immune response appears to be more important. A recombinant yeast-based vaccine expressing the T315I-mutated BCR-ABL antigen was demonstrated to significantly reduce or eliminate BCR-ABL (T315I) leukemia cells from the peripheral blood of immunized animals and extended leukemia-free survival in a murine BCR-ABL + leukemia model [30]. Thus, this may be a potential vaccine for patients with CML.

### WT1 vaccines

WT1 is an oncogenic protein expressed by the Wilms' tumor gene that is overexpressed in the majority of acute myelogenous leukemias (AMLs) and CML. WT1 expression in progenitor cells is minimal or absent, and the limited WT1 tissue expression in adults suggests that WT1 may be a leukemia therapy target. In mice, WT1 vaccines elicit specific immune responses without evidence of tissue damage [31]. Moreover, humoral immune responses against the WT1 protein could be elicited in patients with WT1-expressing hematopoietic malignancies [32]. Therefore, therapeutic vaccines directed against WT1 have the increased expectation that they will be able to elicit and/or boost an immune response to WT1. For example, an imatinib-treated patient with CML who was intradermally administered a WT1 peptide vaccine elicited WT1-specific immune responses and had a resultant reduction in persistent residual disease with the co-administration of imatinib. BCR-ABL mRNA levels were maintained below the detection limit for 8 months beginning at vaccination week 77. The decrease in BCR-ABL mRNA levels was associated with an increase in the frequency of WT1-specific CTLs [33]. These findings indicated that WT1 peptide vaccines may become a safe and cure-oriented treatment for patients with CML who have residual disease despite imatinib treatment.

### Potential LAA vaccines

The BCR-ABL fusion peptide is the predominant antigen in CML, and WT1 is also thought to be important for the identification of leukemia-associated antigens (LAAs) in CML to elicit a specific immune response in patients. However, a more effective and specific immunotherapy with an optimal expression pattern is required for patients with CML, and the identification of additional LAAs is a pivotal step [34,35]. Recently, a number of LAAs that are able to induce specific immune responses were identified in CML including telomerase, PR1, hyaluronan acid-mediated motility (RHAMM), CML-66, CML-28, CML-Ag165, NM23-H2, PPP2R5C, PR3, ELA2, PRAME and a novel epitope derived from the M-phase phosphoprotein 11 protein (MPP11) [34,36-42]. Most of these LAAs have been recognized by human CD8+ T cells. Dendritic cells were DCs pulsed with peptides and then used to generate CTLs. Aurora-A kinase (Aur-A) is a member of the serine/threonine kinase family that regulates the cell division process and has been recently implicated in tumorigenesis. An antigenic 9-amino-acid epitope (Aur-A (207–215): YLILEYAPL), which was derived from Aur-A, is capable of generating leukemia-reactive CTLs in the context of HLA-A*0201. Thus, cellular immunotherapy targeting Aur-A is a promising strategy for leukemia therapy [43].

### DC vaccines

DCs are professional antigen-presenting cells that play a pivotal role in the induction of humoral and cellular immune responses. In the past decade, there has been increasing evidence that tumor antigen-loaded DCs are able to elicit anti-tumor T cell responses. DCs from healthy donors and patients with CML were confirmed to be suitable for clinical application in DC-based immunotherapy protocols in numerous studies.

### CML-derived DC vaccines

In CML, DCs and leukemic cells share common progeny, leading to the constitutive expression of putative tumor antigens, and up to 98% of myeloid DCs generated from peripheral blood mononuclear cells are BCR-ABL positive. The ex vivo differentiation of myeloid leukemic blasts into DCs holds significant promise for use as cellular vaccines. CML-derived DCs (CML-DCs) may have distinct deficiencies such as reduced migration, endocytosis, phagocytosis, antigen processing, DC maturation and cytokine production. CML-DCs are also defective for the processing and presentation of exogenous antigens such as tetanus toxoid. The antigen-processing defects may be a consequence of the reduced capacity of CML-DCs to capture antigens via macropinocytosis or mannose receptors when compared with DCs generated from healthy individuals. This is due to the PKC-induced differentiation associated with the down-regulation of BCR-ABL activity, which raises the possibility that CML-derived DC vaccines will be less effective in presenting leukemia-specific Ags [44]. The DC abnormalities in patients with CML may be abrogated by the proper in vitro stimulation of leukemic DCs [45-47]. However, in 2006, a phase I immunotherapy CML trial demonstrated that DC injections were well tolerated and safe, while no clinical responses was found [48]. The increase in T cell sensitivity to CML-specific stimulation that accompanied active immunization by CML DCs justifies further clinical studies, possibly with modifications such as an increased frequency and number of DC injections. In an additional phase-I/II study, autologous DCs were generated from peripheral blood monocytes and used as a vaccine in patients with chronic phase CML who had not achieved an adequate cytogenetic response after IFN-α or imatinib treatment and demonstrated that T cells that recognize leukemia-associated antigens become detectable [49]. BCR-ABL-expressing DCs appear to inefficiently to induce CML-specific T cell responses resulting from low DC maturation and impaired homing to secondary lymphoid organs. Moreover, BCR/ABL-expressing DCs in the thymus may contribute to CML-specific tolerance induction for specific CTLs [50].

### Ag-loaded “artificial” DC vaccines

Based on the low efficiency of CML-derived DCs for inducing CML–specific CTLs, antigen-loaded DC vaccines may be a strategy to enhance the effects of DC vaccines in CML. A recombinant adeno-associated virus vector encoding the p210 (BCR-ABL) b3a2 variant fusion region with flanking sequences (CWRBA) was constructed and used to express the BCR-ABL fusion region within primary human DCs. CWRBA-transduced DCs elicited cytotoxic CD4+/Th1 and CD8+ responses. Cytotoxicity against a tumor cell line endogenously expressing the p210 (BCR-ABL) b3a2 variant fusion region was also demonstrated. Thus, this developed construct may serve as a candidate vaccine for gene-based, antigen-specific CML immunotherapy [51]. In a small trial, three patients with CML received three series of four administrations of BCR-ABL peptide-pulsed DCs, and all patients developed peptide-specific cellular immune responses with no clinical response. Therefore, the clinical benefits of BCR-ABL peptide-specific vaccinations in CML remain to be determined [52]. Further vaccine development is necessary to increase the clinical effect. Recently, a pilot study was developed to determine whether K562/GM-CSF vaccine immunotherapy could improve clinical responses to imatinib in patients with CML. Nineteen patients with chronic phase CML who achieved at least a major cytogenetic response but had persistent, measurable disease despite one or more years on imatinib were eligible. Each patient was given a series of four vaccines, which was administered in three-week intervals, while remaining on a stable dose of imatinib. In summary, the K562/GM-CSF vaccine appears to improve molecular responses in patients on imatinib, including achieving complete molecular remission [53]. Moreover, the vaccine effect may be enhanced by cytokines such as IL-12, GM-CSF [54].

## Adoptive T-cell therapy

Adoptive antigen-specific immunotherapy is one of the best approaches for tumor immunotherapy. Antigen-specific CTLs can directly kill tumor cells while ignoring the host immune status.

### Donor leukocyte infusion

Donor leukocyte infusion (DLI) after marrow transplantation has induced lasting remission in the majority of patients with CML in hematological or cytogenetic relapse. This effect also provided the first direct evidence of the graft-versus-leukemia (GVL) effect after allogeneic bone marrow transplantation (BMT) [55]. There is a strong GVL of allogeneic stem cell transplantation (allo-HSCT) effect due to the elimination of tumor cells by alloimmune effector lymphocytes, which is used for the prophylaxis of leukemia relapse and improvement of transplantation outcomes [56,57].

DLI can be safely administered to patients relapsing after unmodified, unrelated allografts, exerting an antileukemic effect that is sufficiently potent for reversing the accelerated disease phase. Moreover, DLI was also used in patients with CML who were refractory to IFN-α or imatinib, lacked donors for allo-HSCT, and received autotransplants followed by the infusion of ex vivo costimulated autologous T cells [58]. However, graft-versus-host disease (GVHD) is a major complication of DLI. One of the methods to control GVHD may be improved using suicidal gene-modified T cells for DLI, allowing T-cell elimination if severe GVHD develops [59]. If the GVL effect could be separated from GVHD through the adoptive transfer of selected T cells that recognize leukemia-specific antigens or minor histocompatibility antigens (miHAs), which are expressed predominantly on hematopoietic cells, it may preclude an attack on normal tissues. The GVL effect induced by DLI is thought to be mediated by clonally expanding T cells with different TCR Vβ repertoires in different patient groups such as Vβ16 or Vβ21 [16,60]. The transient proliferation of the limited number of T cells detected in the peripheral blood 3–5 months after DLI probably reflects the GVL response against CML cells and may serve as a marker for the appearance of the GVL effect induced by DLI [60].

Recently, in a BCR-ABL1 transduction/transplantation mouse model for studying the DLI mechanisms in MHC-matched, miHA-mismatched allogeneic chimeras with CML-like leukemia, the GVL effect from DLI could be identified, and the GVL effect is directed against miHAs shared by normal and leukemic stem cells, which are predominantly mediated by CD8+ T cells with minor contributions from CD5-splenocytes including natural killer cells. These results define a physiological model of CML adoptive immunotherapy that will be useful for investigating the GVL cellular and molecular mechanisms [61].

### CTL

Although DLI has been effective in patients with CML relapsing after allo-HSCT, efforts to augment GVL and immune reconstitution have been limited by GVHD. One approach for augmenting GVL has been to infuse ex vivo-generated T cells with defined specificities. Different alternative approaches for specific adoptive immunotherapy with CTLs targeted to different CML-associated LAAs were reported including CD8+ T cells from donors vaccinated against a single miHA expressed by leukemia cells, PRAME CTLs, donor derived b2a2-specific and b3a2-specific T cells, and Aur-A-specific CTLs [43,62-66].

Recently, infusion using in vitro-generated donor T cells reactive against peptides derived from CML-associated antigens was reported in a trial with 14 patients with CML, who received conditioning therapy followed by CD34+ selected HSCT from matched siblings or unrelated donors. Donor-derived mature DCs generated in vitro from CD14+ monocytes were loaded with human leukocyte Ag-restricted peptides derived from PR1, WT1, and/or BCR-ABL and used to repetitively stimulate donor CD8+ T cells in the presence of IL-2 and IL-7. Stimulated T cells were infused 28, 56, and 112 days after transplantation. Thirteen patients survived and 7 remain in molecular remission (median follow-up: 45 months). Importantly, all patients receiving CD8+ T cells displaying marked cytotoxic activity in vitro and detectable peptide-reactive CD8+ T cells during follow-up have not experienced GVHD or relapse. These results reveal that the prophylactic infusion of allogeneic CD8+ T cells that are reactive against peptides derived from CML-associated antigens is a safe and promising therapeutic strategy [67].

In general, the most effective specific CTLs from DLI were αβ + T cells that were HLA restricted. Increasing data demonstrate that γδ + T cells play a critical role in anti-leukemia after stem cell transplantation. Moreover, γδ + T cells activated by phosphoantigens or agents that induce their accumulation within cells e.g., zoledronate, may represent a promising strategy for the design of a novel and highly innovative immunotherapy capable of overcoming imatinib resistance. Recently, an animal study has demonstrated that Vγ9Vδ2 + T cells recognize trogocytosis and efficiently kills imatinib-sensitive and imatinib-resistant CML cell lines pretreated with zoledronate, and its cytotoxicity is largely dependent on the granule exocytosis- and, partially, TRAIL-mediated pathways, was TCR-mediated and required isoprenoid biosynthesis by zoledronate-treated CML cells. Importantly, Vγ9Vδ2 + T cells from patients with CML can be induced by zoledronate to develop antitumor activity against autologous and allogeneic zoledronate-treated leukemia cells, both in vitro and when transferred into immunodeficient mice in vivo. Therefore, this study may indicate that the intentional activation of Vγ9Vδ2 T cells by zoledronate may substantially increase their antileukemia activities and represent a novel strategy for CML immunotherapy [68].

### T cell receptor gene modified T cells

Adoptive transfer of antigen-specific T cells is an attractive immunotherapy method for hematological malignancies and cancer. The difficulty of isolating antigen-specific T cells for individual patients limits the more widespread use of adoptive T cell therapy. The demonstration that cloned T cell receptor (TCR) genes may be used to produce T cell populations of desired specificity offers new opportunities for antigen-specific T cell therapy. In hematologic malignancies, TCR specificity for WT1 peptides, a peptide from the EB virus, and a DLBCL-associated antigen were transferred into donor T cells and displayed specificity against tumor cells expressing specific LAAs [69,70]. The first trial in humans demonstrated that TCR gene-modified T cells specific for the MART-1 protein persisted for an extended time and reduced tumor burden in some patients. The Strauss HJ research team isolated high avidity CTLs specific for a WT1-derived peptide presented by HLA-A2 and cloned the TCR α and β genes of a WT1-specific CTL line. The genes were inserted into retroviral vectors for the transduction of human peripheral blood T cells from patients with leukemia and normal donors. While the treatment of leukemia-bearing NOD/SCID mice with T cells modified with a WT1-specific TCR eliminated leukemia cells in the bone marrow of most mice, treatment with T cells transduced with a TCR with irrelevant specificity did not diminish the leukemia burden. The development of new TCR gene constructs holds great promise for the safe and effective delivery of TCR gene therapy for the treatment of malignancies [69]. The first phase I clinical trial with TCR-modified CTLs was performed as a melanoma therapy in 2006 [71], and WT1- and BCR-ABL-specific TCR-modified CTLs have potential for CML immunotherapy in future.

Alternatively, chimeric TCRs (chTCRs) comprising single-chain variable fragments (scFvs) of murine antibodies and human signaling molecules were used to redirect the specificity of autologous or allogeneic T cells and were developed as novel therapeutic agents for CML treatment. Wang D et al. engineered a scFv from the CMA1 hybridoma cell line, which produces monoclonal antibodies that are specific against CML. The genes encoding the heavy and light chain variable regions were amplified from CMA1 cDNA, and a humanized chTCR was constructed. The novel hchTCR specific for CML cells can be expressed, normally presented on the cell surface, and may be used to redirect human T cells [72].

## Conclusions and future investigation

Data from vaccination and adoptive T cell immunotherapy for CML have indicated that the immune system may contribute to disease control in CML. Preliminary data from specific TCR-modified T cells against CML will have to be validated in well-designed clinical trials with cells generated via reproducible methods and in accredited structures working according to good manufacturing practices.

Based on the PD-1/PD-L1 expression feature in CML cells and specific T cells for CML, blocking the PD-1/PD-L1 interaction may restore the function of CML-specific CTLs and represent a novel therapeutic approach for CML.

## Competing interests

The authors declare that they have no competing interests.

## Authors’ contribution

The concept of this paper was devised by YQL. YQL, CL and CAS contributed to the intellectual input of the paper. All authors read and approved the final manuscript.
